# Intramedullary nailing of the femur and the systemic activation of monocytes and neutrophils

**DOI:** 10.1186/1749-7922-6-34

**Published:** 2011-10-31

**Authors:** Falco Hietbrink, Leo Koenderman, Luke PH Leenen

**Affiliations:** 1 Department of Surgery, University Medical Center Utrecht, the Netherlands; 2 Department of Respiratory Medicine, University Medical Center Utrecht, the Netherlands

**Keywords:** Trauma, ARDS, neutrophils, tissue damage

## Abstract

**Background:**

Trauma such as found patients with femur fractures, induces a systemic inflammatory response, which ranges from mild SIRS to ARDS. Neutrophils (i.e. PMN) play an important role in the pathogenesis of this inflammatory condition. Additional activation of PMNs during intramedullary nailing (IMN) is thought to act as a second immunological hit. Damage control orthopedics has been developed to limit this putative exacerbation of the inflammatory response. The hypothesis is tested that IMN exacerbates systemic inflammation, thereby increasing the risk for ARDS.

**Methods:**

Thirty-eight trauma patients who required IMN for femur fracture were included. The development of SIRS and ARDS was recorded. Blood samples were taken prior and 18 hours after IMN. Inflammatory response was analyzed by changes in plasma IL-6 levels, monocyte (HLA-DR) and PMN phenotype (MAC-1 and responsiveness for the innate immune stimulus fMLP in the context of active FcγRII).

**Results:**

Plasma IL-6 was significantly enhanced in severely injured patients compared to patients with isolated femur fractures and matched controls (P = 0.005; P = 0.018). This enhanced inflammatory tone was associated with a lower percentage HLA-DR positive monocytes (P = 0.002). The systemic PMN compartment was activated, characterized by an increased MAC-1 expression and a significantly decreased sensitivity for the innate stimulus fMLP Interestingly the PMN compartment was not affected by IMN.

**Conclusions:**

Multitrauma patients were characterized by a marked activation of the systemic inflammatory response, associated with a systemic activation of the monocyte and PMN compartments. IMN particularly affected the monocyte arm of the systemic innate immune system.

## Introduction

ARDS (Acute Respiratory Distress Syndrome) is a frequent complication after trauma. Although mortality rates has been reduced over the last decade by improved treatment strategies and modalities, morbidity rates remain high, as the incidence of ARDS has only slightly decreased [1]. Several risk factors have been identified for the development of ARDS, such as intramedullary osteosynthesis/nailing (IMN) of a femoral fracture, massive blood transfusion and thoracic injury [2]. When IMN is performed in the presence of these risk factors, the incidence of ARDS can be over 40%[3,4]. In this case, IMN is seen as a second hit.

Systemic inflammation is key in the development of ARDS. The amplitude of this systemic response is often measured by plasma IL-6 levels. However, systemic activation of the cellular innate immune system is essential in the development of ARDS [5]. When extravasation of polymorphonuclear granulocytes (i.e. PMNs or neutrophils) is blocked or animals are depleted of PMNs, no ARDS occurs after a sufficient insult [6]. In addition, in patients with sepsis, circulating HLA-DR negative monocytes were identified, which point at a pro-inflammatory profile, as described previously. These cells are thought to contribute to additional tissue damage [7]. The role of these cells during IMN has not been investigated yet.

This etiological study was designed to test the hypothesis whether IMN contributes to a more pronounced systemic inflammation, characterized by a change phenotype of cells of the innate immune system. This hypothesis was tested in 2 subgroups of patients with different injury severity (isolated femur fracture and femur fracture in multitrauma).

## Patients and methods

### Patients

Forty-five trauma patients were included in this study. They were admitted to the Department of Traumatology, University Medical Center Utrecht with a fracture of the femur, which required primary or secondary intramedullary nailing. Exclusion criteria were age < 16 years or > 80 years and patients with an altered immunological status (e.g. use of corticosteroids or chemotherapy). The local ethical committee approved the study and written informed consent was obtained from all patients or their spouses in accordance to the protocol.

### Clinical parameters and sampling

The Injury Severity Score and APACHE II Score were calculated on admission. During admission the occurrence of pulmonary complications (i.e. acute lung injury [ALI] or acute respiratory distress syndrome [ARDS]) were assessed according to their clinical criteria as determined in the consensus conference [8].

Recently, we have shown that the extent of systemic inflammation of innate immune cells can be visualized by measuring the expression of activation markers on blood PMNs [9]. The most sensitive marker turned out to be the responsiveness of active FcγRII (CD32) on PMN's for the innate immune stimulus fMLP [9,10]. The most commonly used marker is MAC-1 (CD11b), which peaks between 6 and 18 hours after insult (i.e. trauma or surgery)[11]. In contrast to PMN's, changes in activation of the systemic monocyte compartment can be determined by analyzing the percentage of circulating HLA-DR positive monocytes [7].

Blood samples were taken at two distinct time points: one hour prior to IMN and 18 hours after the intramedullary nail was introduced. To investigate the influence of IMN, patients were stratified by isolated femur fracture and femur fractures in multitrauma. Patients were compared with healthy, age and gender matched controls as described previously (see Table 1)[9].

**Table 1:** Patient demographics.

	Median (+ range)
**Number of patients (n)**	38
**Male/Female (n)**	22/16
**Age (years)**	30 (16-80)
**Injury Severity Score**	13 (9-43)
**- Femur fracture (n = 23)**	10 (9-19)
**- Multitrauma (n = 15)**	29 (16-43)
**APACHE II Score**	5 (0-24)
**Time on ICU (days)**	0 (0-60)
**Time on ventilation (days)**	0 (0-55)
**Packed red blood cells before first blood sample (units)**	0 (0-22)
**Fresh frozen plasma before first blood sample (units)**	0 (0-20)
**Trauma mechanism (n)**	
- **MVA**	29
- **Fall of height**	8
- **Direct impact**	1
**Complications (n)**	
- **No SIRS symptoms**	14
- **SIRS**	17
- **ALI/ARDS**	7

### Materials

For analysis of PMN receptor expression by flowcytometry the following monoclonal antibodies were commercially purchased: FITC-labeled IgG1 negative control (clone DD7, Chemicon, Hampshire, United Kingdom), RPE-labeled IgG2a negative control (clone MRC OX-34, Serotec, Dusseldorf, Germany) and RPE-labelled CD11b (clone 2LPM19c, DAKO, Glostrup, Denmark). An antibody, which recognizes an active FcyRII/CD32 (designated FcyRII*), is manufactured at the Department of Pulmonary Science at the University Medical Center Utrecht (MoPhab A27, UMCU, Utrecht, The Netherlands)[12,13].

For analysis of monocyte HLA-DR expression by flowcytometry the following monoclonal antibody was commercially purchased: FITC-labeled HLA-DR (YE2/36-HLK, Serotec, Dusseldorf, Germany).

### Pmn and monocyte receptor expression

The inflammatory status of a patient can be assessed by analyzing the expression of active FcyRII (FcyRII*) on PMNs in the peripheral blood [9]. A low expression of fMLP induced FcyRII* correlates with increased inflammation. This approach has been validated in a previous study [9]. The expression of fMLP induced FcyRII* was compared with a more common activation marker MAC-1 (CD11b)[14].

Blood was collected in a vacutainer^® ^with sodium heparin as anticoagulant, cooled immediately and kept on ice during the whole staining procedure. The analysis of the PMN receptor expression was started within two hours after the blood sample was obtained. The expression of the above mentioned markers was measured as described previously [9]. Expression of active FcγRII by FITC-labeled MoPhab A27 was measured after 5 minutes of stimulation of whole blood at 37°C with N-formyl-methionyl-leucyl-phenylalanine (fMLP 10^-6^M) to evaluate the responsiveness of the cells for a bacterial derived activating agonist. After stimulation, the samples were put on ice again and analyzed.

Blood samples were stained with fluorescein isothiocyanate (FITC) directly labeled antibodies (MoPhab A27) as described previously [9]. The expression of CD11b and HLA-DR were performed according the recommendations of the manufacturer. In short, directly labeled antibodies were added 1:20 to whole blood and incubated for 60 minutes on ice. After incubation, the red cells were lysed with ice-cold isotonic NH_4_Cl. After a final wash with PBS2+ (phosphate buffered saline with added sodium citrate (0,38% wt/vol) and isotonic pasteurized plasma proteins (10% vol/vol), the cells were analyzed in a FACScalibur Flowcytometer (Becton & Dickenson, Mountain view. CA). The PMNs and monocytes were identified according to their specific side-scatter and forward-scatter signals. Data from individual experiments are depicted as histograms of fluorescence intensity in arbitrary units (AU) or summarized as the median channel fluorescence (MCF) of at least 10000 events.

### Interleukin-6

IL-6 was determined using a human IL-6 sandwich ELISA (Endogen, Pierce Biotechnology, IL, United States) according to the procedures prescribed by the manufacturer. Detection limit of this ELISA was 5 pg/ml.

### Statistical Analysis

All data were analyzed using SPSS version 15.0 software (The Apache Software Production 2008, Chicago, Illinois). Results are expressed by medians + range. Statistical analysis was performed using a non-parametric Mann Whitney U Test for two groups and a Kruskall Wallis H test for multiple comparisons. Paired analysis (before and after surgery) was performed using Wilcoxon Signed Ranks test. Statistical significance was defined as p < 0.05.

## Results

### Demographics

A total of 45 patients fulfilled the inclusion criteria in a period of 1 year. Of these 45 patients, 3 patients were missed due to logistical restrictions, 2 patients underwent external fixation initially, but did not receive conversion to intramedullary osteosynthesis, 1 patient did not give consent and in 1 patients sampling was flawed. Thus, 38 patients were adequately followed up (84%). Their median ISS was 13 (range 9-43) and their median APACHE II Score was 5 (range 0-25) at admission. Intramedullary nailing was performed either directly or in a staged damage control approach. Seven patients developed ALI/ARDS, which indicates an adequate patient selection. Further demographics are listed in Table 1.

### Types Of Injury

**Figure 1:**
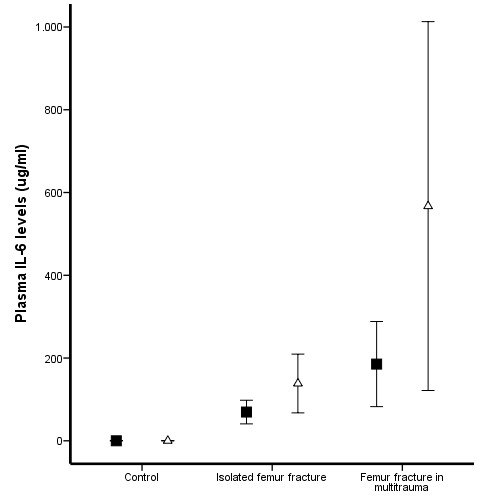
**Plasma IL-6 levels**. Multitrauma patients demonstrated increased levels of plasma IL-6 compared to patients with isolated femur fracture (P = 0.018) or matched controls (P = 0.005). *Pre-operative *IL-6 levels ("black square") were significantly increased in patients who developed respiratory failure (P < 0.001). Eighteen hours after intramedullary nailing ("open triangle"), plasma IL-6 levels were significantly increased in patients with isolated femur fractures (P = 0.030), but not in multitrauma patients (P = 0.515), which could be due to insufficient power.

**Figure 2:**
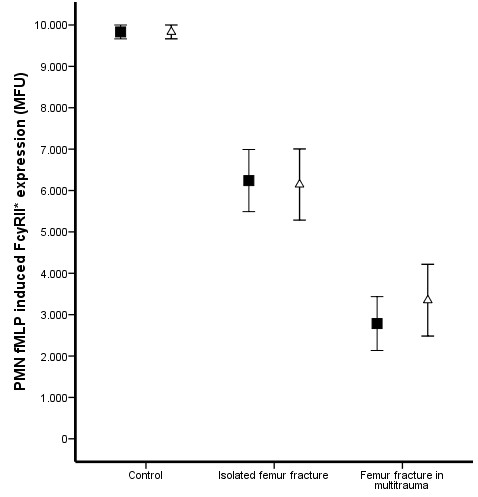
**PMN fMLP induced FcyRII expression**. Multitrauma patients demonstrated decreased expression of fMLP induced FcyRII on PMNs compared to patients with isolated femur fracture (P = 0.004) or matched controls (P < 0.001). *Pre-operative *fMLP induced FcyRII* ("black square") was more decreased in patients who developed ARDS (P < 0.001). Eighteen hours after intramedullary nailing ("open triangle"),fMLP induced FcyRII* did not change compared to pre-operatively.

**Figure 3:**
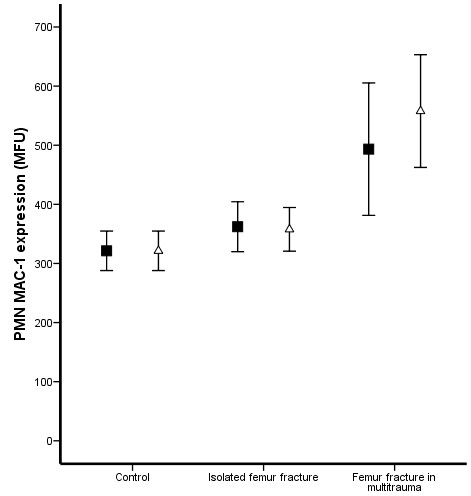
**PMN MAC-1 expression**. No statistical significant increased MAC-1 expression was seen in multitrauma patients. In addition, no increased pre-operative expression ("black square") was demonstrated in patients who developed respiratory failure and no difference was seen 18 hours after intramedullary nailing ("open triangle").

**Figure 4:**
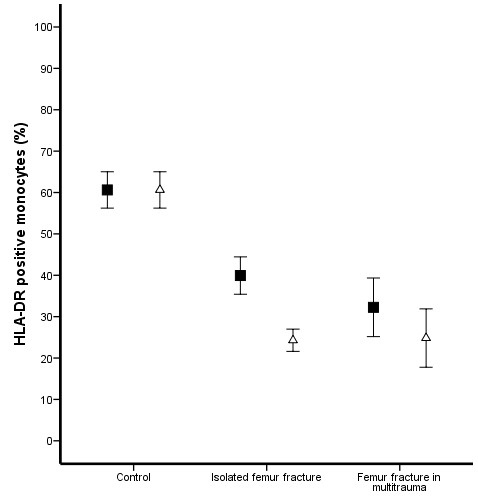
**HLA-DR positive monocytes**. The percentage HLA-DR positive monocytes was decreased in all patients compared to controls (P = 0.002). The p*re-operative *("black square") lowest percentage was seen in patients who developed respiratory failure (P = 0.002). Eighteen hours after intramedullary nailing ("open triangle"), a further decrease in HLA-DR positive monocytes was seen in patients with isolated femur fracture (P < 0.001) and multitrauma patients (P = 0.047).

Prior to surgery, multitrauma patients demonstrated increased levels of plasma IL-6 (Figure 1) and a decreased expression of fMLP induced FcγRII* on PMNs (Figure 2) compared to patients with an isolated femur fracture (P = 0.005 and P < 0.0001 respectively) and matched control donors (P = 0.018 and P = 0.004 respectively). In contrast, MAC-1 expression (Figure 3) and the percentage HLA-DR positive monocytes (Figure 4) did not demonstrate a difference between multitrauma patients and patients with isolated femur fractures. The percentage HLA_DR positive monocytes was decreased in all patients, compared to matched control donors (P = 0.002). There was no significant correlation between plasma IL-6 levels and cellular markers, indicating that the measured markers identify different aspects of the systemic inflammatory response.

### Symptoms Of Systemic Inflammation During Follow-Up

Seven patients developed respiratory failure and fulfilled the ALI/ARDS criteria, whereas 17 patients only fulfilled the SIRS criteria during the 48 hours after IMN. *Pre-operative *IL-6 levels were significantly increased in patients who developed respiratory failure (P < 0.001). *Pre-operative *expression of fMLP induced FcγRII* on PMNs (P < 0.001) and the percentage of HLA-DR positive monocytes (P = 0.002) was lower in patients with more severe inflammatory response. In contrast, MAC-1 expression did not demonstrate a significant difference in patients with a more severe inflammatory response.

### Impact of intramedullary nailing

Eighteen hours after intramedullary nailing, plasma IL-6 levels were significantly increased in patients with isolated femur fractures (P = 0.030), but not in multitrauma patients (P = 0.515, Figure 1). The activation markers of PMNs (fMLP induced FcγRII* and MAC-1) did not change after intramedullary nailing in either patients with isolated femur fracture or multitrauma patients (Figure 2 and 3). In contrast, the percentage HLA-DR positive monocytes decreased significantly in both patient groups (P < 0.001 of isolated femur fractures and P = 0.047 for multitrauma patients, Figure 4).

## Discussion

This study confirms that multitrauma patients have a significant inflammatory response measured by plasma levels of IL-6 and PMNs phenotype. Furthermore, patients who developed ALI/ARDS demonstrated severe systemic inflammation measured by plasma IL-6 levels and PMN activation markers. This study is thereby comparable with previous studies which measured plasma cytokine levels and PMN phenotype. In addition, we measured PMN activation towards the innate stimulus fMLP. Active inside-out control of PMNs towards fMLP was significantly decreased in patients with more severe injuries. However, with this sensitive measurement, no additional activation of PMNs occurred after IMN of femur fractures, in either patients with isolated femur fractures or multitrauma patients.

Trauma induces inflammation and severe inflammation has been related to the development of ALI/ARDS [15]. It has been demonstrated that PMNs play an essential role in the pathophysiology of ALI/ARDS, whereas the roles of cytokines (such as IL-6) and monocytes are less clear, because these cytokines often have multiple target cells and different functions. IL-6 levels have often been used for their prognostic importance, but no causal pathophysiological relation has been identified [16,17]. It is true that more trauma results in more systemic inflammation and thus in more cytokine release. However, IL-6 does not cause damage to the pulmonary endothelium. Products produced by PMNs cause this damage and our data support the importance of PMNs. Severe trauma results in an altered PMN phenotype patients who developed ARDS demonstrated the most activated PMNs. In addition, our study suggest a role for monocytes as well in the pathophysiology of ALI/ARDS. Monocyte HLA-DR expression was decreased in multitrauma patients, indicating a more pro-inflammatory type of monocytes which has been suggested previously to contribute to the tissue damage during a systemic inflammatory response.

We next studied the impact of intramedullary nailing on the systemic inflammatory response. Additional inflammation caused by surgery is seen as additional trauma and has been considered as a possible risk factor for organ failure such as ARDS [18]. Much to our surprise the increased damage caused by IMN only partly induced changes in the systemic inflammatory response (only monocyte HLA-DR expression in patients with isolated femur fractures). Most striking was the absence of additional PMN activation after intramedullary nailing. This lack of change in PMN phenotype during IMN is in line with suggestions from a previously published report [19]. In that cohort, no increase was seen in MAC-1 expression on PMNs after bilateral femur fracture fixation. Thus, the extend of PMN activation appears mainly determined by the severity of initial trauma and is apparently not altered by intramedullary nailing. In contrast, plasma IL-6 levels and monocyte HLA-DR were significantly altered by intramedullary nailing. Thus, an impact of the surgical procedure can be measured by cytokines and the monocyte compartment.

The blood samples were taken 1 hour prior to IMN and 18 hours after IMN, regardless of the interval between trauma and surgery. Although this affects the reproducibility of the results, it reflects daily care practice. 18 hours after IMN the peak of plasma IL-6 levels will be passed (max at 6 hours post-operatively), but the changes in PMN phenotype will be most defined. PMN phenotype behaved similarly in all patients, therefore, 38 patients were sufficient to state the conclusion.

Because we analyzed the functional phenotype of PMNs and monocytes, more information was obtained than merely static phenotypes. The inflammatory cellular response deficit to the development of ARDS appears to be mainly determined by the initial injuries and not the additional insult by IMN.

## Competing interests

The authors declare that they have no competing interests.

## Authors' contributions

FH sampled the patients, performed the analysis and drafted the manuscript, LK supported in the sample analysis and revised the manuscript. LL participated in the design of the study and revised the manuscript. All authors read and approved the final manuscript.
